# Young scientist perspective—microbiology trainees and social media: making science go viral during a pandemic

**DOI:** 10.1093/femsmc/xtab001

**Published:** 2021-04-07

**Authors:** Kait F Al, Scarlett Puebla-Barragan, Gregor Reid, Jeremy P Burton

**Affiliations:** Department of Microbiology and Immunology, University of Western Ontario, London, Canada; Department of Microbiology and Immunology, University of Western Ontario, London, Canada; Department of Microbiology and Immunology, University of Western Ontario, London, Canada; Lawson Health Research Institute, London, Canada; Department of Microbiology and Immunology, University of Western Ontario, London, Canada; Lawson Health Research Institute, London, Canada

**Keywords:** scientific communication, social media, young scientists

## Abstract

This perspective looks at how social media has become even more critical since the pandemic and provides tips on how to market research outputs from a trainee viewpoint better.

## INTRODUCTION

Communication of results is what gives research a real impact as a scientist. The teaching of scientific communication to trainees in disciplines such as microbiology has typically revolved around delivering robust peer-review focused outputs. However, research findings are often disseminated through television interviews, lectures, or even Instagram posts. In 2020, the world was turned on its head in more ways than one. Now more than ever, it is crucial to protect and promote accurate scientific communication, and more than any other platform, social media has become an increasingly utilized tool in this process. Social media has altered the trajectory of scientific communication in terms of its reach (e.g. worldwide, regardless of race, education, gender, lingual and socioeconomic disparities), pace (immediate, in comparison to the lengthy journal review process) and capacity (virtual conferences between hundreds to thousands of people during quarantine). How this movement shifts young scientists' future and scientific communication will be substantial, but it remains uncertain like many things in the current climate. Although once considered scientifically distasteful, it may be time to provide learning resources and mentorship to trainees to maximize their scientific outputs in an accurate and meaningful way to meet the needs of a new global learning environment.

### Inclusive scientific communication

The movement towards equity, diversity and inclusivity has been one of the central issues to erupt this year, in a tempest fueled in part by social media. Unfortunately, the sciences are not an exception to the lack of inclusivity, not only in terms of ethnicity or place of origin but also in terms of gender and differing physical abilities. Social media has enlightened many of us to the different lived experiences of people in the sciences, and in particular, academia. Often, only the voices of a select group of individuals are heard in STEM (Higher Education Today 2019), and this lack of representation of minority groups negatively impacts their participation in the sciences. Thanks to social media, people who identify as part of an underrepresented minority now have a platform to raise their voices and share their opinions and achievements; this helps to level the ground, making everyone visible.

**Figure 1.**
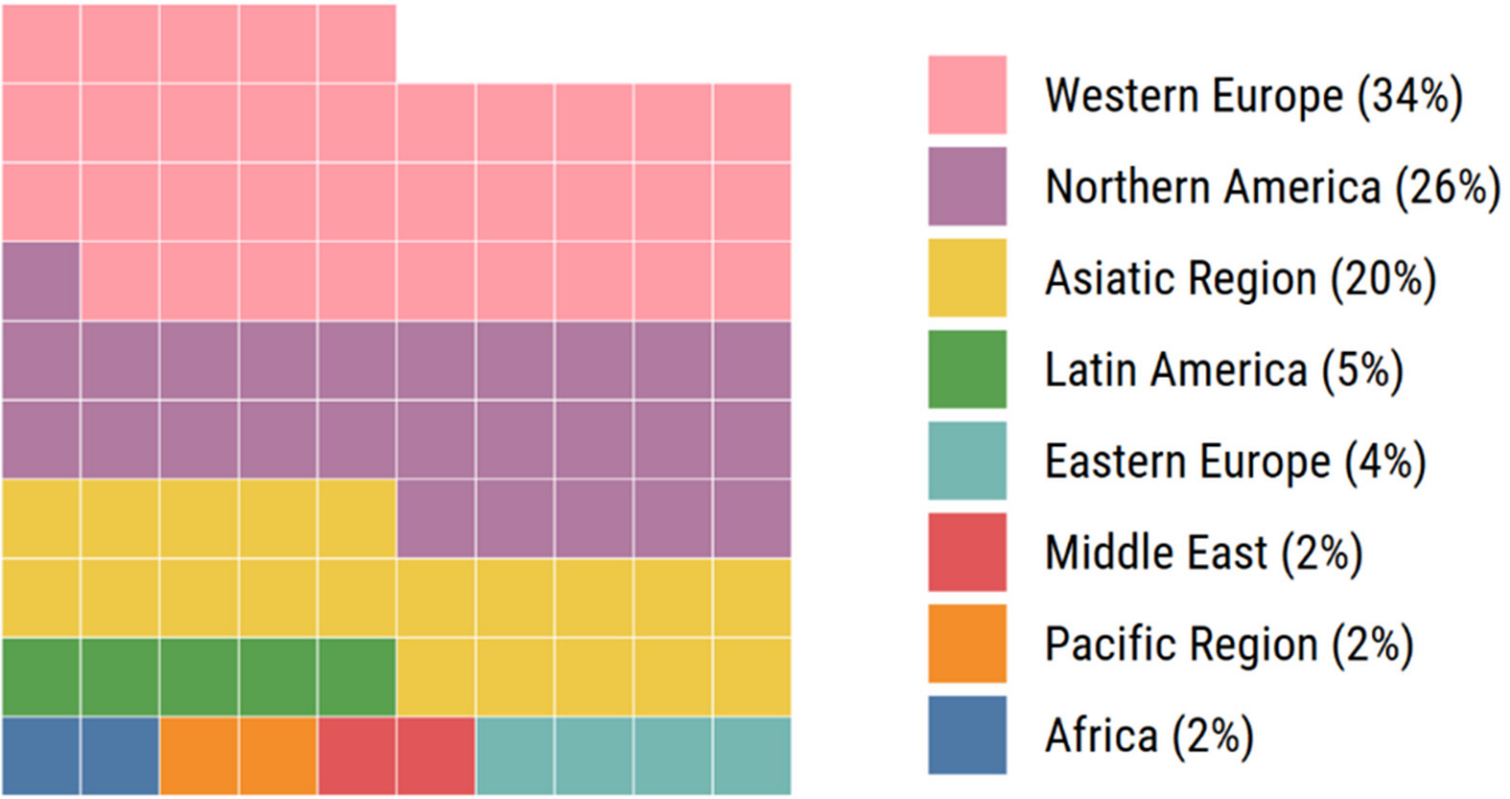
Geographical distribution of scientific production in the area of microbiology. Waffle chart shows the proportion of citable documents produced by each geographical region from 1996 to 2019 according to SciMago (Scimago 2020,November 20). Chart created in R version 4.0.2.

Another advantage of the current communication technologies is their potential to link the entire scientific world (Guerrero-Medina *et al*. 2013). It is possible to network and to have scientific discussions with people from almost anywhere, providing and receiving feedback, regardless of geographical location. More specifically, Twitter has become the preferred platform for many academics to exchange ideas and network. Access to these platforms allows underrepresented minorities to shed light on obstacles they may uniquely face, as well as a means to network and showcase their work and discoveries. This dialogue alone is a powerful step towards breaking barriers and stereotypes, inspiring new generations of scientists with diverse backgrounds. Nowadays, most scientific findings in microbiology are made in a few geographical regions (Scimago November 20 2020; as shown in Fig. 1 ). As such, people from other countries tend to migrate for an opportunity to partake in this work (Franzoni *et al*. 2012; Aceituno-Aceituno *et al*. 2018). Social media enables scientists to collaborate with peers in their place of origin after they emigrate, enriching scientific diversity, and communicating their work in a relevant and accessible way to their culture.

**Figure 2.**
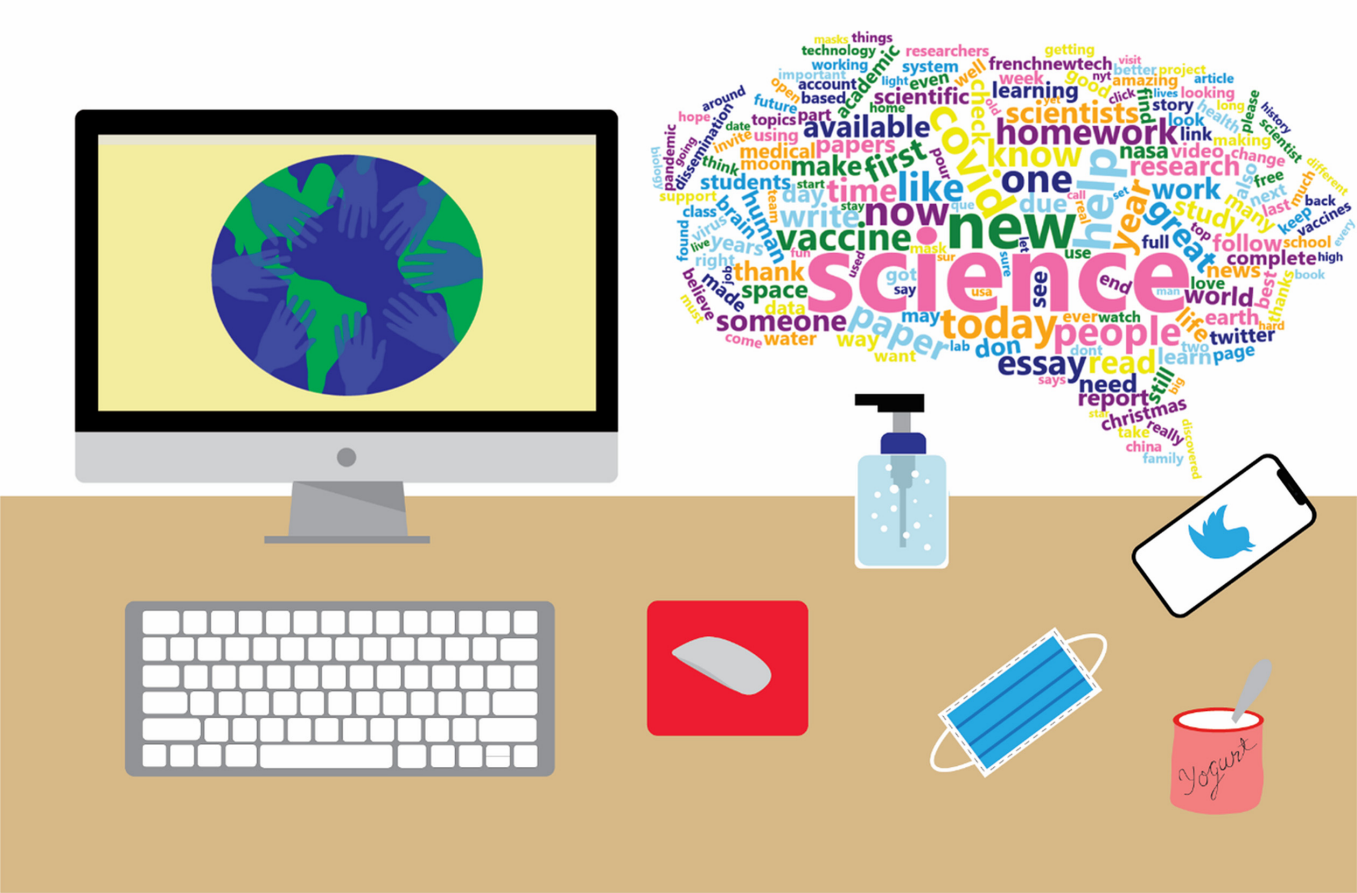
The workplace of a microbiologist during a global pandemic. Visual representation of the ‘new normality’ and how scientists have adapted during a time were socialization has dramatically changed, using technology to help bring the world and scientific conversation together. The word cloud shows the most common terms used in tweets that contain the hashtag ‘#science’ during the month of December 2020. Word analysis was performed using the ‘rtweet’ package (Kearney 2019) in R version 4.0.2.

The collaboration that has arisen from social media should now be translated into the ‘real world.’ Once we return to face-to-face interactions, it is our responsibility as scholars to ensure that all voices are equally heard and to make sure the ground is not only levelled in the ‘online world.’

### The evolution of virtual networking

This pandemic has necessitated the cancellation of the majority of large, in-person gatherings. Unfortunately, this has included most scientific conferences, which have instead transitioned to virtual events. Networking is a primary goal for many conference attendees, and in the absence of handshaking, a newfound emphasis has been thrust upon digital networking. Navigating this transition successfully through both its advantages and demerits has far-reaching implications for young scientists.

For example, during virtual conferences, both the method and message of disseminated research can be fundamentally changed. A previously interactive in-person poster session may now be replaced by viewing pre-recorded talks and poster files, occasionally introducing live Q&A sessions. An obvious outcome will be the development of improved digital presentation skills. However, by virtue of being online, the research is now forever public. An unfortunate side effect of this transition may be that researchers are less inclined to share nascent ideas or work that is not directly in line for publication, although a more positive alternative path may be the more rapid sharing of unpublished data beyond preprints by younger scientists. Another example comes from live-tweeting during conferences (both virtual and in-person), which has gained popularity over recent years. This practice can connect like-minded thinkers (i.e. potential collaborators and job opportunities) and opens the exchange of information beyond just the presentation's immediate audience. However, the resulting information is not peer-reviewed and can lead to the potential dissemination of misinformation.

As fields evolve and face-to-face interactions are limited, digital communication as a career tool affords superior accessibility and inclusion. Whether it be through a conference's app, social media, academic networking sites or a wealth of other potential avenues, it is clear that now more than ever, digital engagement is crucial to successful remote work and to building meaningful connections, online or otherwise.

### Enhancing old school (preprints, blogging and communication teams)

While journals attempt to speed the publishing processes up, they are still mostly reliant on a pool of unpaid reviewers that have little incentive for rapid turnaround. However, it is unlikely that the gold standard of peer-reviewed publication of science will be replaced in the near future. There are some waves being made with platforms such as ‘Review Commons’ which offer impartial objective reviews for manuscripts and preprints which may preclude further specific review when submitted to one of the affiliated journals (Review Commons 2021). This is limited to a selection of journals at present, but one could imagine how this service could greatly streamline the publishing process. Another way to elevate traditional scientific outputs beyond social media and traditional journals is to deposit a preprint to an online repository. These rapidly published manuscripts are not typically peer reviewed. However, they are useful for works in highly competitive fields or where a budget does not conform with the ever-increasing journal publication fees. While interest in the peer-reviewed article may have waned when it is finally published (since the scientific community has already seen the findings in preprint format), this approach is increasing in popularity. It has been a crucial tool in releasing the lightning-speed COVID-19 research of 2020 (Majumder and Mandl 2020).

There are several other opportunities for publicizing that may be overlooked by scientists. One such avenue is blogging, which can disseminate one's findings at different levels of appreciation from scientific to lay audiences. Some of the larger publishing groups (Nature Publishing Group, and Science, for example) have such sites established to complement their peer-reviewed content. Another avenue is to promote work through academic-focused social networking sites such as ResearchGate and Academia.edu (ResearchGate 2021; Academia 2021). Navigating the many different routes of scientific communication may be aided by employing professionals. Most academic institutions have a communications team of some description, an asset to employ upon the release of significant research findings. One advantage of having research promoted through their channels is that it will likely reach more people faster by accessing their significant online following. Typically, members of communication teams are experts at repackaging material to where it will be of interest to the general media and lay public, making it more likely for other media outlets to pick up your research.

### A changing perception of microbes

The COVID-19 pandemic and the accompanying lockdowns forced many people indoors, increasing screen time and social media consumption (Fig. 2). How this has affected physical and mental health is likely far-reaching and beyond this piece's scope, but the impact on public opinion of the microbial world is another significant concern.

Although the divergent pandemic responses exhibited globally have demonstrated attitudes ranging from horror to blasé, an overarching cultural shift towards germaphobia may be occurring. At present, the emphasis on hand hygiene and surface sterilization is necessary given the global havoc wreaked by this virus. However, this practice is not altogether harmless and instead paints the microbial world with a broadly fear-inducing brush. The recent surge in sterilization has not only increased chemical exposures and accidental poisonings from cleaning supplies across North America (Chang *et al*. 2020; Collie 2020). Furthermore, it may lead to alterations to the beneficial microbiome of human skin (Vandegrift *et al*. 2017; Zapka *et al*. 2017). How long-lived the panic-driven opinions remain after the immediate threat of COVID-19 is contained will help shape our microbiomes in the future.

Similarly, as research and the roll-out of an effective vaccine against COVID-19 continues, this topical dialogue may shape public opinion of herd immunity and vaccination. Recent studies have cited vaccine hesitancy stemming from the small yet vocal anti-vaccination movement (Megget 2020). In this way, the fear and misinformation spread during this pandemic may be enabling the broadcast and dissemination of more extremist views (Megget and June 2020; Phillips 2020; Vaccine Confidence Project 2020). To combat this changing public climate of sterilization, infection, and vaccination, effective and accessible scientific communication to the masses from scientific leaders within the field is necessary.

Beyond a negative view of microbes, there is an opposite emerging interest in some microorganisms' many benefits and essential roles on human health. These curiosities range from the protection from respiratory infections by taking probiotics (Baud *et al*. 2020), trying to understand the associations between the gut microbiome and COVID-19 (Zuo *et al*. 2020; Yeoh *et al*. 2021), and mainstream interest in preparing fermented foods such as sourdough bread or kombucha as part of popular ‘quarantine activities.’ It is now the job of microbiologists to capitalize on this public interest for research and start paying closer attention to beneficial microorganisms instead of solely focusing on those that cause disease.

Contrastingly to the more dire responses to this pandemic, passionate interest and awareness of science have arisen throughout the population. Microbiology has never been more present in the daily lives of people worldwide, and we are all witnessing the application of the scientific method in real-time. The general public has fostered a novel and deep appreciation for the scientific method, since the pandemic has given them a glimpse of the work behind the scenes in designing and testing protocols, treatments, and vaccines. We must harness this momentum going forward and maintain trust in science during uncertain times. As such, it is essential to embrace all evolving methods of scientific communication; the need for academics to stay connected during these unprecedented times has accelerated our ability to adapt and take maximum advantage of what technology offers.

It is now clear that the tools for scientific dissemination are changing and that even post COVID-19, some of the ways that we engage with others will be altered indefinitely. Once the world crisis is over, we should take a retrospective look and identify which parts of the new normality are worth keeping and have propelled scientific advancement into this new era of scholarly and scientific communication. We need to both utilize and teach our trainees methods that allow a broader, more diverse audience, and give a voice to previously underrepresented groups to shape the future direction of science for the better.

## ABOUT THE AUTHORS

Dr Kait Al is a postdoctoral fellow who studies the microbiota associated with urological conditions. She undertook her graduate and undergraduate studies at the University of Western Ontario (Western).

Scarlett Puebla-Barragán is a senior Ph.D. student interested in probiotics and the metabolome associated with urogenital health. She undertook her undergraduate training at the Monterrey Institute of Technology and Higher Education (Tec de Monterrey) in Mexico and is completing her graduate studies at Western in the Department of Microbiology and Immunology. Dr Burton and Dr Reid are the respective supervisors.

## Supplementary Material

xtab001_Supplemental_File
