# Supplementary material for: Young scientist perspective—microbiology trainees and social media: making science go viral during a pandemic
Source: FEMS Microbes. 2021 Apr 7;2:xtab001. doi: 10.1093/femsmc/xtab001 (PMC10117738; doi:10.1093/femsmc/xtab001)
Supplement: xtab001_Supplemental_File [file xtab001_supplemental_file.docx]

Peer review comments and author response for FEMSMC-2021-005

Reviewer 1: Guillaume Thibault

Comments to the author:

In this commentary manuscript, the authors discuss how the current pandemic reshaped ways the scientific community interact and share knowledge.

Overall, the authors' arguments are clear, logical, and easy to understand. The manuscript is well written. I only have two minor comments that the authors can consider if they wish to do so.

(1) The authors stated "An unfortunate side effect of this transition may be that researchers are less inclined to share nascent ideas or work that is not directly in line for publication" (Page 3, line 43-47). Is is also correct to think that it might drive sharing unpublished data beyond preprint, especially among young scientists?

(2) The authors stated "However, it is unlikely that the gold standard of peer-reviewed publication of science will be replaced in the near future. Another" (Page 4, line 20-23). Some initiatives have been recently implemented to rock the boat. Review Commons and eLife (publish than review) for instance.

Author response:

Thank you Editors,

We have made all of the suggested small changes suggested.
